# P-1316. Influenza Antiviral Prescribing Practices for Outpatients with an Acute Respiratory Illness and at Increased Risk for Influenza-Associated Complications pre- and post-COVID-19 pandemic — United States, 2016–2022

**DOI:** 10.1093/ofid/ofae631.1496

**Published:** 2025-01-29

**Authors:** Jessie R Chung, Sascha Ellington, Ashley Price

**Affiliations:** Centers for Disease Control and Prevention, Atlanta, GA; CDC, Atlanta, Georgia; Centers for Disease Control and Prevention, Atlanta, GA

## Abstract

**Background:**

Early antiviral treatment (< 2 days after illness onset) can reduce the risk of influenza-associated complications and is recommended for outpatients at increased risk. However, in previous influenza seasons, antivirals have been underprescribed. We describe influenza antiviral prescribing practices for outpatients from 2016-2022.

**Methods:**

We analyzed influenza antiviral prescription data from 5 US Influenza Vaccine Effectiveness Network sites pre-pandemic, 2016-2017 through 2019-2020, and post-pandemic, 2021–2022 (2020-2021 season not included due to low influenza circulation). Patients enrolled were aged ≥6 months presenting with an acute respiratory illness with a new or worsening cough ≤7 days duration. All had respiratory specimens collected for influenza RT-PCR testing. Patients at increased risk for severe outcome included those aged < 2 years, ≥ 65 years, and those with at least one underlying medical condition. Electronic medical records were examined to determine whether antivirals were prescribed within 7 days after enrollment.

**Results:**

Among 21,483 patients at increased risk enrolled in the US Flu VE Network between 2016-2017 and 2021-2022, 8% (1,852) were prescribed antivirals within 7 days. In pre-pandemic seasons, the average proportion of those prescribed antivirals was 9.5% overall and lowest during the 2019-2020 season (6%); post-pandemic, this proportion decreased to 1%. Among confirmed influenza cases, 40% (874/2210) of those at increased risk who presented for care < 2 days after illness onset were prescribed antivirals across all 5 seasons. Similarly, during the pre-pandemic seasons the average proportion of patients prescribed antivirals was 40% with the lowest percentage observed during the 2019-2020 season (26%). This proportion continued to trend downward post-pandemic (21%).

**Conclusion:**

Over the last several seasons, influenza antiviral medications have been infrequently prescribed, including among those at increased risk for severe illness who present early in their illness. Efforts to increase appropriate antiviral prescribing may reduce influenza-associated complications among outpatients presenting early for care.

**Disclosures:**

**All Authors**: No reported disclosures

